# Supplementary material for: Ultralong purely organic aqueous phosphorescence supramolecular polymer for targeted tumor cell imaging
Source: Nat Commun. 2020 Sep 16;11:4655. doi: 10.1038/s41467-020-18520-7 (PMC7494876; doi:10.1038/s41467-020-18520-7)
Supplement: Supplementary file 1 — Supplementary Information [file 41467_2020_18520_MOESM1_ESM.pdf]

Supplementary Information

Ultralong Purely Organic Aqueous Phosphorescence Supramolecular Polymer for Targeted Tumor Cell Imaging

Wei-Lei Zhou[1], Yong Chen[1], Qilin Yu[1,2], Haoyang Zhang[1], Zhi-xue Liu[1], Xian-Yin Dai[1], Jing-Jing Li[1], Yu Liu[1]*

[1]College of Chemistry, State Key Laboratory of Elemento-Organic Chemistry, Nankai University, Tianjin 300071, P. R. China

[2]Key Laboratory of Molecular Microbiology and Technology, College of Life Sciences, Nankai University, Tianjin 300071, China.

*E-mail: yuliu@nankai.edu.cn

Supplementary Discussion

Synthesis of BrBP–NH_2.

3–Bromopropan–1–amine hydrobromide (0.23 g, 1.00 mmol) was added to a solution of 4–(4–bromophenyl)pyridine (0.47 g, 2.00 mmol) in acetonitrile (50 ml). The solution was heated under reflux for 2 h, during which time a large amount of precipitate formed. The reaction mixture was allowed to cool to room temperature and then filtered, and the obtained solid was washed thoroughly with acetonitrile to afford BrBP–NH_2 as a pale yellow solid (0.15 g, 67%). ^{1}H NMR (400 MHz, D_2O, 25 °C) δ 8.88 (d, J = 7.0 Hz, 2H), 8.35 (d, J = 6.9 Hz, 2H), 7.88–7.82 (m, 4H), 4.75–4.71 (m, 2H), 3.20–3.14 (m, 2H), 2.53–2.40 (m, 2H). ^{13}C NMR (100 MHz, D_2O, 25 °C) δ 156.09, 144.22, 132.78, 131.35, 129.66, 126.69, 125.21, 57.66, 36.17, 28.32.

HRMS (ESI) for $C_{14}H_{16}Br_2N_2$: calcd. $[M–HBr–Br]^+$: 291.05, found: 291.05.

Synthesis of HA–BrBP.

1–Ethyl–3–(3–dimethylaminopropyl)carbodiimide (1.68 g, 0.875 mmol) and *N*–hydroxysulfosuccinimide sodium salt (1.9 g, 0.875 mmol) were added to a solution of sodium hyaluronate (*Mw* = 250,000, 1.00 g, 0.53 μmol) in phosphate buffered saline (0.1 M, pH 7.2, 30 mL). The mixture was stirred at 25 ℃ for 30 min, and then BrBP–NH_2 (1.14 g, 0.25 mmol) in phosphate buffered saline (10 mL) was added. After stirring at room temperature for 24 h, the solution was dialyzed against excess deionized water for 5 days. Freeze–drying afforded HA–BrBP as a white powder. ^{1}H NMR (400 MHz, D_2O, TMS) δ 1.98 (s, 3 H, H of methyl group of HA), 2.84–4.60 (m, H of HA and the alkyl chain of BrBP–NH_2), 8.84, 8.32, 7.81 (H of the benzene ring of BrBP–NH_2). Using the single–point method and the integrated peak area of the benzene ring and the HA backbone in the NMR spectrum, we determined the degree of substitution to be 3.5%.

a

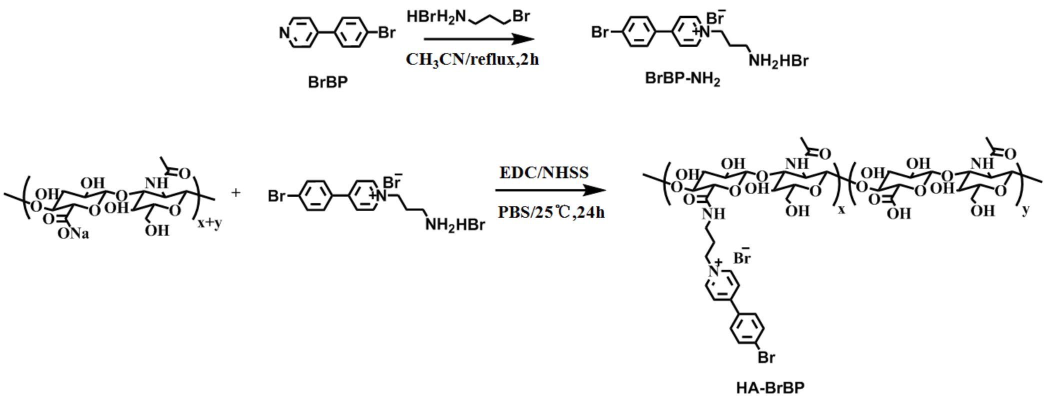

b

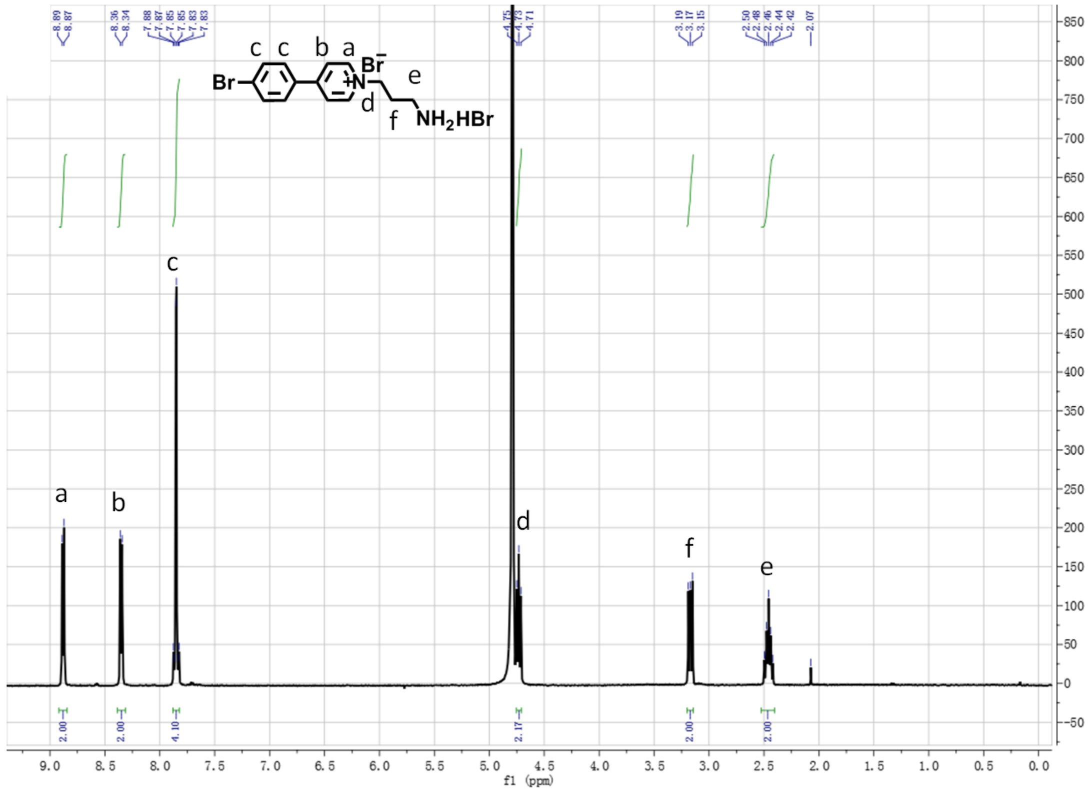

c

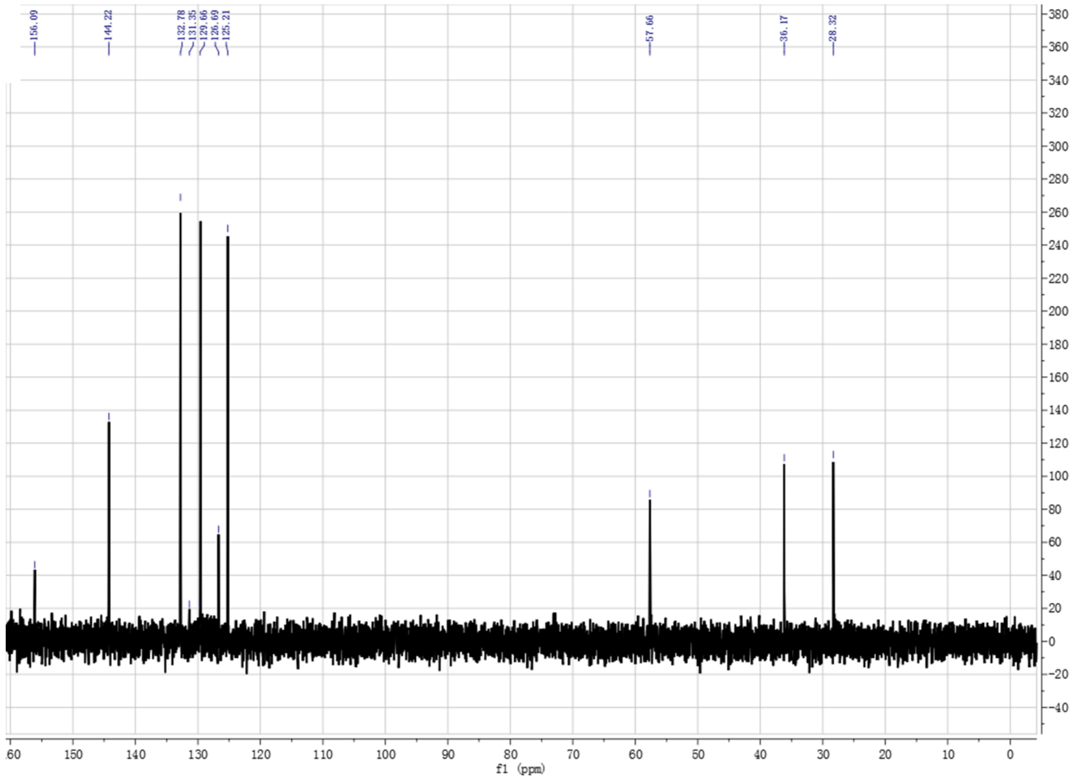

d

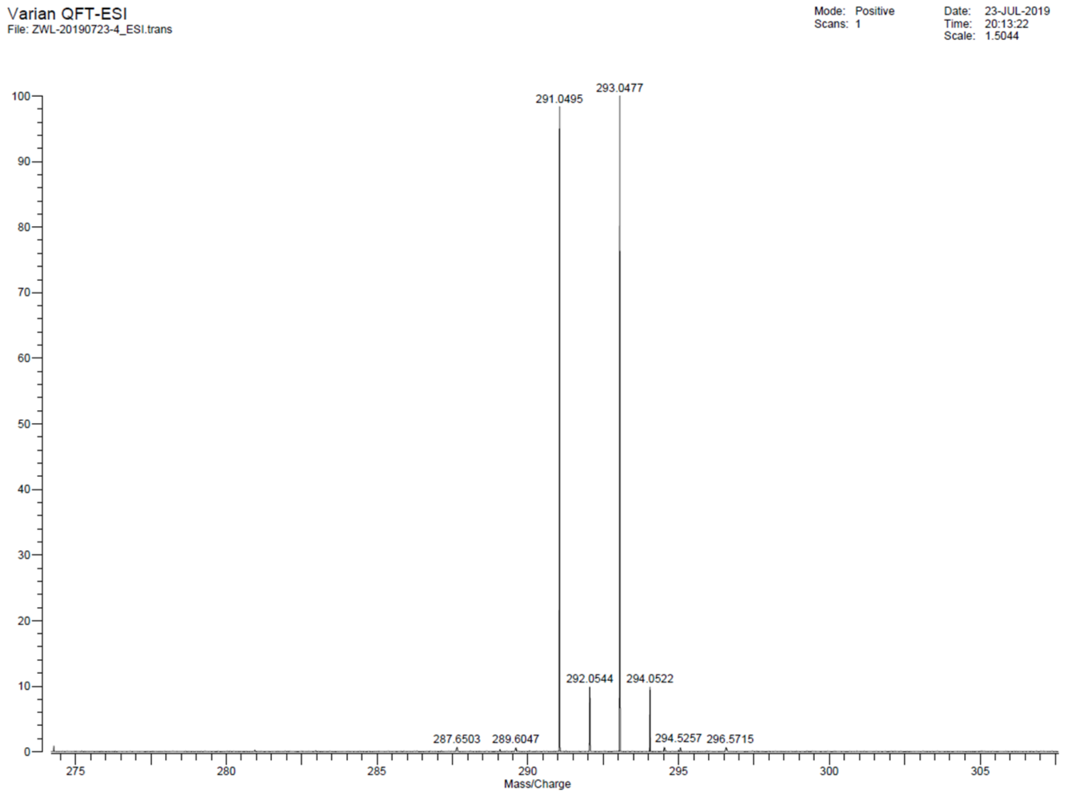

Supplementary Figure 1. Characterization of BrBP–NH$_2$. (**a**) Synthetic route of BrBP–NH$_2$; (**b**) ^{1}H NMR spectrum of BrBP–NH$_2$ (400 MHz, D_2O, 25 ℃); (**c**) ^{13}C NMR spectrum of BrBP–NH$_2$ (100 MHz, DMSO–d_6, 25 ℃); (**d**) ESI mass spectrum of BrBP–NH$_2$.

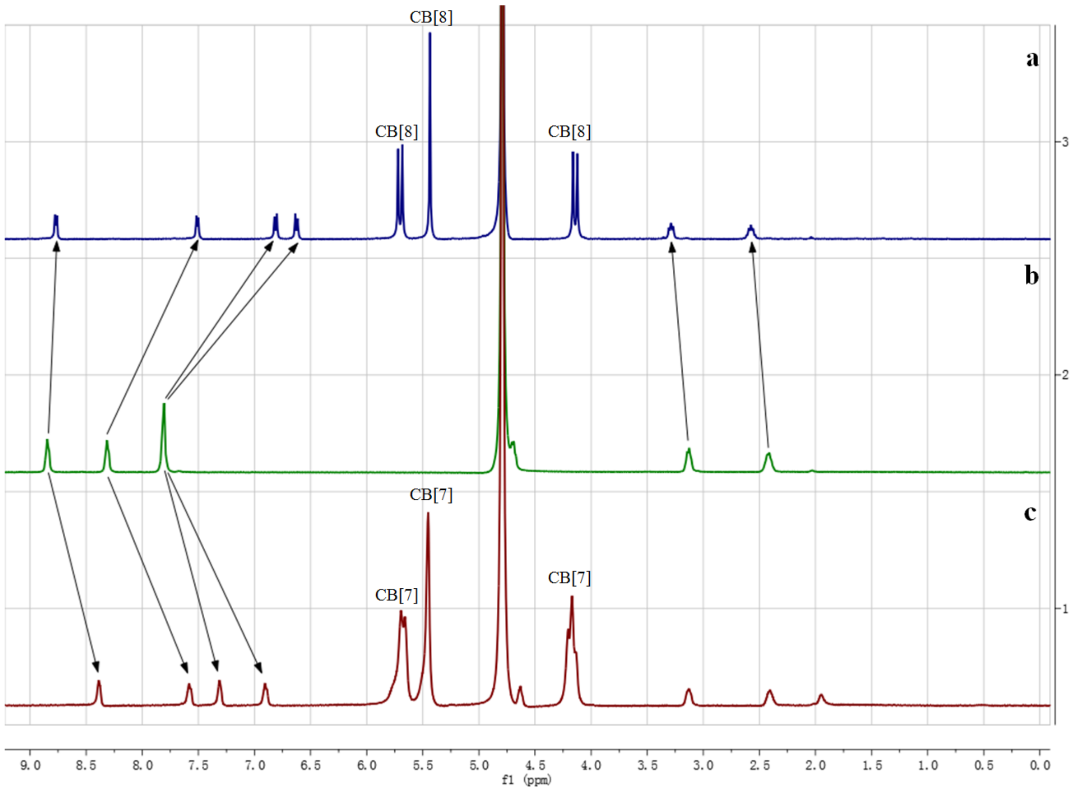

Supplementary Figure 2. ^{1}H NMR spectra of CB[8]/BrBP–NH_2, BrBP–NH_2, and CB[7]/BrBP–NH_2. ^{1}H NMR spectrum (D_2O, 400 MHz, 298 K) of (**a**) CB[8]/BrBP–NH_2 complex, (**b**) BrBP–NH_2 and (**c**) CB[7]/BrBP–NH_2 complex.

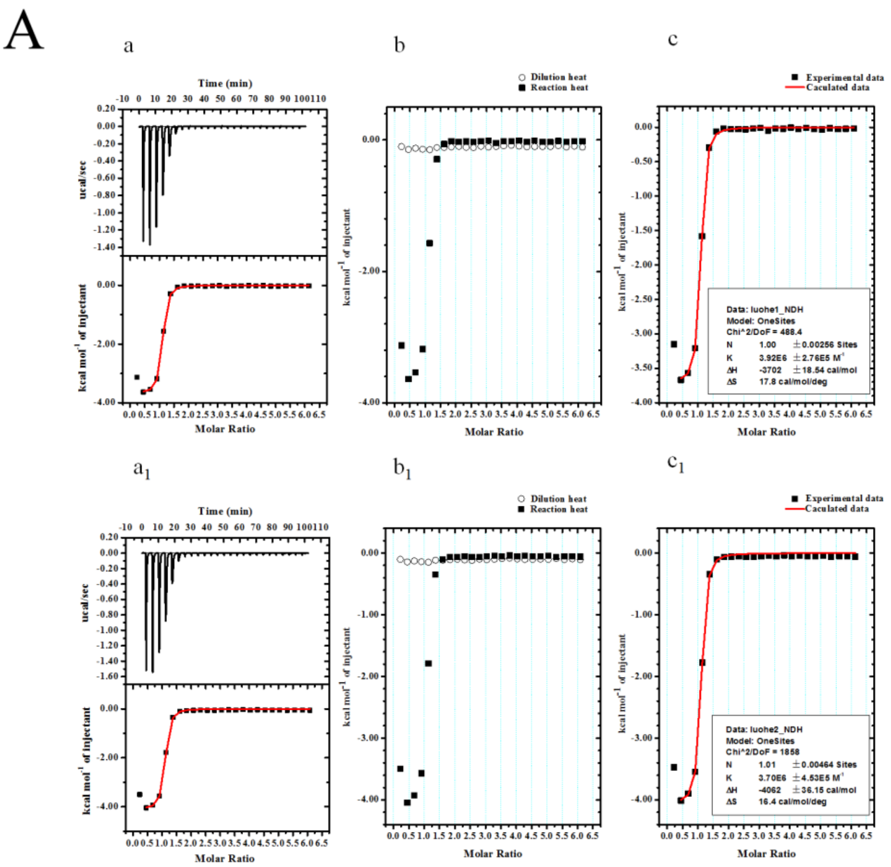
A
a
b
c
Time (min)
ucal/sec
kcal mol-1 of injectant
Molar Ratio
Dilution heat
Reaction heat
Experimental data
Caculated data
Data: luohe1_NDH
Model: OneSites
Chi^2/DoF = 488.4
N 1.00 ±0.00256 Sites
K 3.92E6 ±2.76E5 M-1
ΔH -3702 ±18.54 cal/mol
ΔS 17.8 cal/mol/deg
a1
b1
c1
Data: luohe2_NDH
Model: OneSites
Chi^2/DoF = 1858
N 1.01 ±0.00464 Sites
K 3.70E6 ±4.53E5 M-1
ΔH -4062 ±36.15 cal/mol
ΔS 16.4 cal/mol/deg

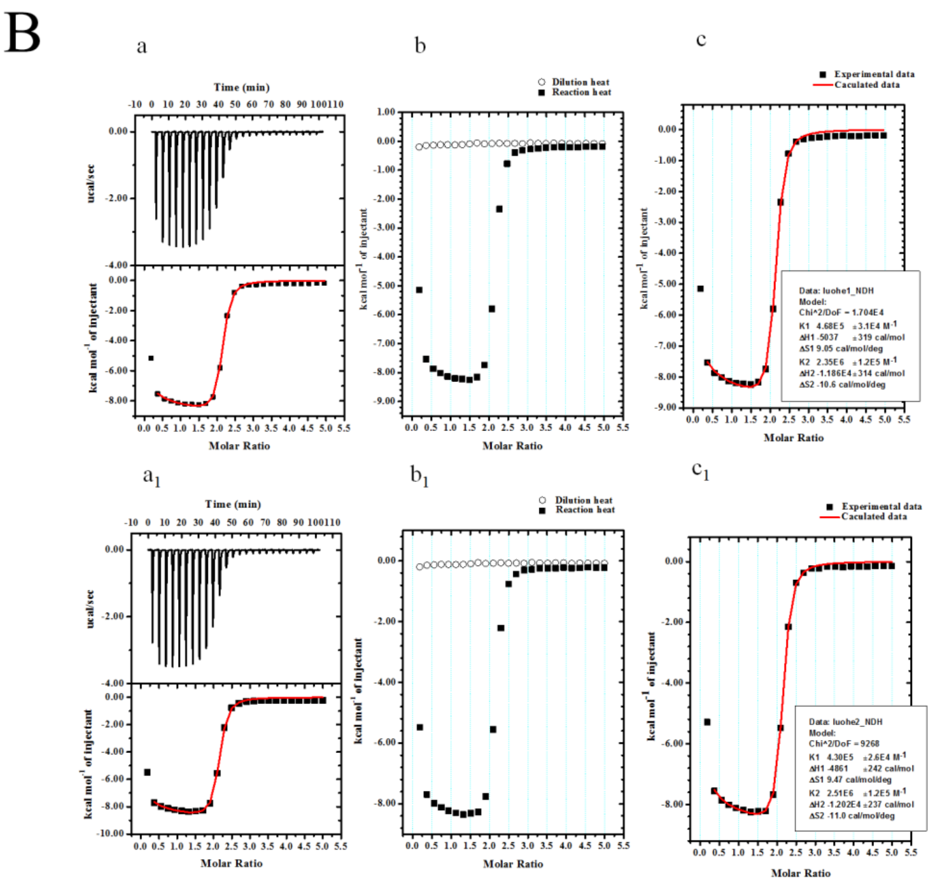

Supplementary Figure 3. ITC data for CB[7]/BrBP–NH_2 and CB[8]/BrBP–NH_2 complexation. (**A**) Calorimetric titrations in aqueous solution for sequential 25 injections (10 μL per injection) of BrBP–NH_2 solution (1.25 mM) injecting into CB[7] solution (0.048 mM): (a) raw data and apparent reaction heat; (b) heat effects of the dilution and of the complexation reaction; (c) "Net" heat effects fitted using the "one set of binding sites" model. The thermodynamic data in CB[7]/BrBP–NH_2 complexation were obtained as K_S = (3.81 ± 0.22) × 10^6 M^{-1}, $-\Delta H$ = (1.63 ± 0.15) × 10^4 J·mol^{-1}, $T\Delta S$ = (2.13 ± 0.18) × 10^4 J·mol^{-1}, and $-\Delta G$ = (3.76 ± 0.01) × 10^4 J·mol^{-1}, respectively; (**B**) Calorimetric titrations in aqueous solution for sequential 25 injections (10 μL per injection) of BrBP–NH_2 solution (1.28 mM) injecting into CB[8] solution (0.04 mM): (a) raw data and apparent reaction heat; (b) heat effects of the

dilution and of the complexation reaction; (c) "Net" heat effects fitted using the "1:2 sequential binding sites" model. The thermodynamic data in CB[8]/BrBP–NH_2 complexation were obtained as $K_{S,1}$ = (4.49 ± 0.19) × 10^5 M^{-1} , $K_{S,2}$ = (2.43 ± 0.08) × 10^6 M^{-1} , –ΔH = (7.07 ± 0.003) × 10^4 J·mol^{-1} , TΔS = (1.92 ± 0.01) × 10^3 J·mol^{-1} , and – ΔG = (6.87 ± 0.005) × 10^4 J·mol^{-1} , respectively.

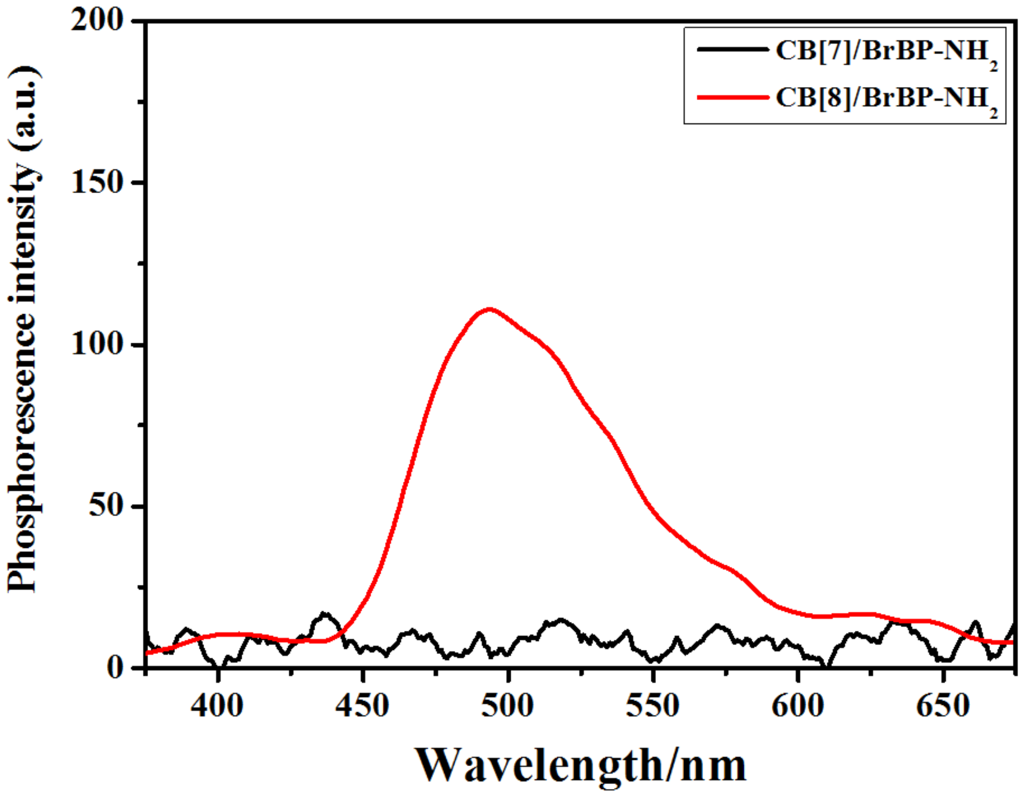

Supplementary Figure 4. Phosphorescence contrast spectra (delayed 0.2 ms) of CB[7]/BrBP–NH_2 and CB[8]/BrBP–NH_2. The phosphorescence spectra (delayed by 0.2 ms) of CB[7]/BrBP–NH_2 (black) and CB[8]/BrBP–NH_2 (red) (BrBP–NH_2 = 0.5 mM, CB[7] = 0.5 mM, CB[8] = 0.25 mM) in water (25 ℃, λ_{ex} = 320 nm) (Ex. Slit = 5 nm, Em. Slit = 5 nm).

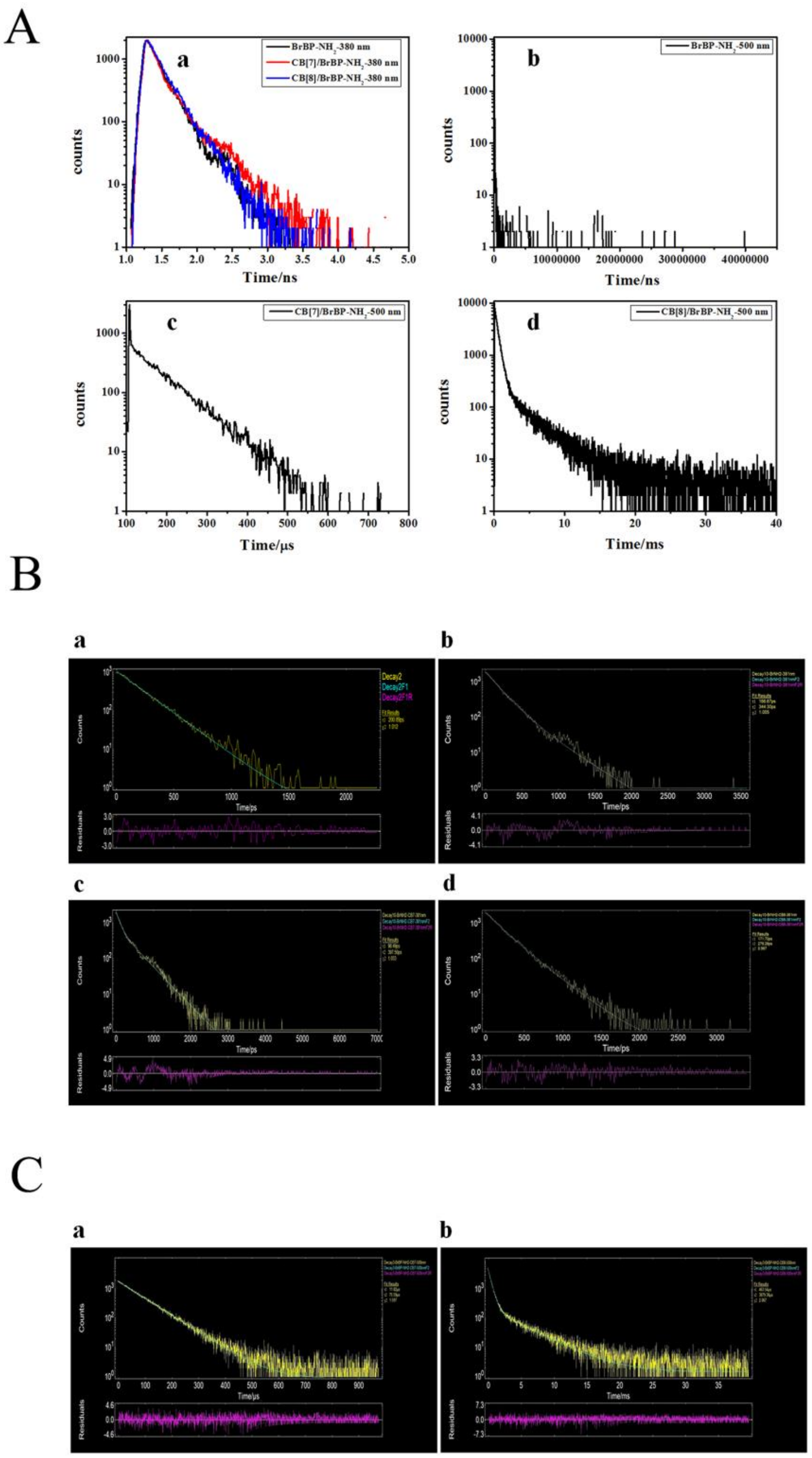

Supplementary Figure 5. Fluorescence and phosphorescence lifetime contrast curves for CB[7]/BrBP–NH$_2$ and CB[8]/BrBP–NH$_2$. (**A**) (a) Fluorescence decay curves of BrBP–NH$_2$, CB[7]/BrBP–NH$_2$ and CB[8]/BrBP–NH$_2$ at 380 nm at 298 K. Phosphorescence decay curves

of (b) BrBP–NH_2; (c) CB[7]/BrBP–NH_2 and (d) CB[8]/BrBP–NH_2 at 500 nm at 298 K. (BrBP–NH_2 = 0.5 mM, CB[7] = 0.5 mM, CB[8] = 0.25 mM); (**B**) Fluorescence lifetime decay fitting curve of (a) HA–BrBP (b) BrBP–NH_2, (c) CB[7]/BrBP–NH_2 and (d) CB[8]/BrBP–NH_2 measured for 380 nm at 298 K. (BrBP–NH_2 = 0.5 mM, CB[7] = 0.5 mM, CB[8]=0.25 mM); (**C**) Phosphorescence lifetime decay fitting curve of (a) CB[7]/BrBP–NH_2 (b) CB[8]/BrBP–NH_2 measured for 500 nm at 298 K**.** (BrBP–NH_2 = 0.5 mM, CB[7] = 0.5 mM, CB[8] = 0.25 mM).

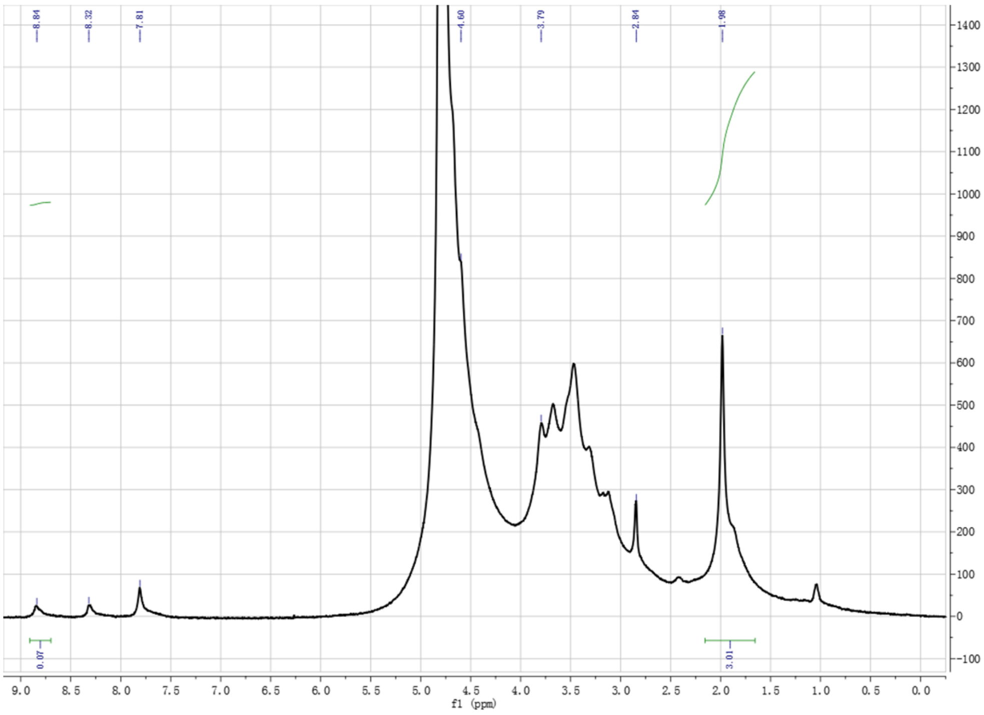

Supplementary Figure 6. ^{1}H NMR spectrum of HA–BrBP. ^{1}H NMR spectrum of HA–BrBP in D_2O at 298 K.

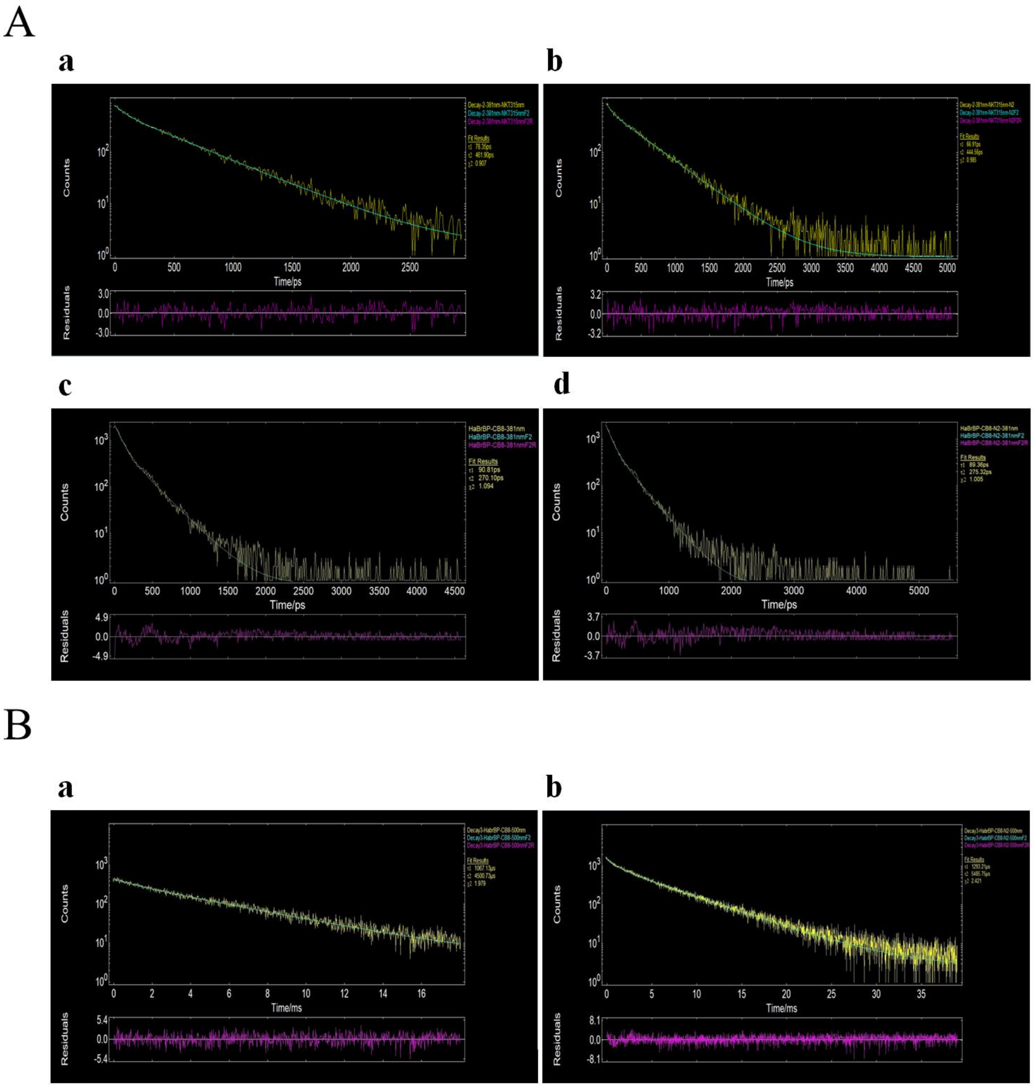

Supplementary Figure 7. Fluorescence and phosphorescence lifetime decay fitting curves for CB[7]/HA–BrBP and CB[8]/HA–BrBP. (**A**) Fluorescence lifetime decay fitting curve of (a) CB[7]/HA–BrBP, (b) CB[7]/HA–BrBP/N_2, (c) CB[8]/HA–BrBP and (d) CB[8]/HA–BrBP/N_2 measured for 380 nm at 298 K. ([BrBP] = 0.5 mM, [CB7] = 0.5 mM, [CB8] = 0.25 mM); (**B**) Phosphorescence lifetime decay fitting curve of (a) CB[8]/HA–BrBP (b) CB[8]/HA–BrBP/N_2 measured for 500 nm at 298 K. ([BrBP] = 0.5 mM, [CB8] = 0.25 mM).

Supplementary Table 1. RTP lifetimes reported in the literature.

structure	Pτ	literature
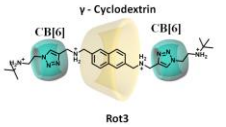	2.5 ms	Ref.12
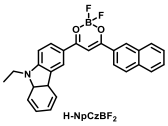	29.0 μs	Ref.24
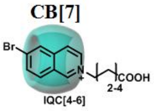	127.38 μs	Ref.28
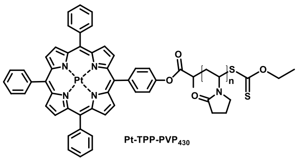	7.96 μs	Ref.22
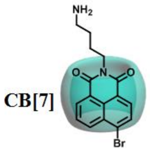	400 μs	Ref.30
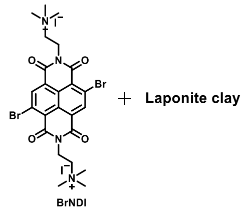	385 μs	Ref.6
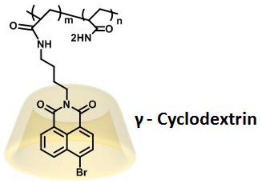	320 μs	Ref.29
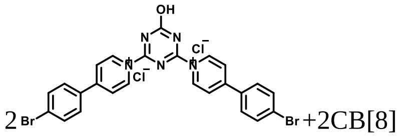	190 μs	Ref.31

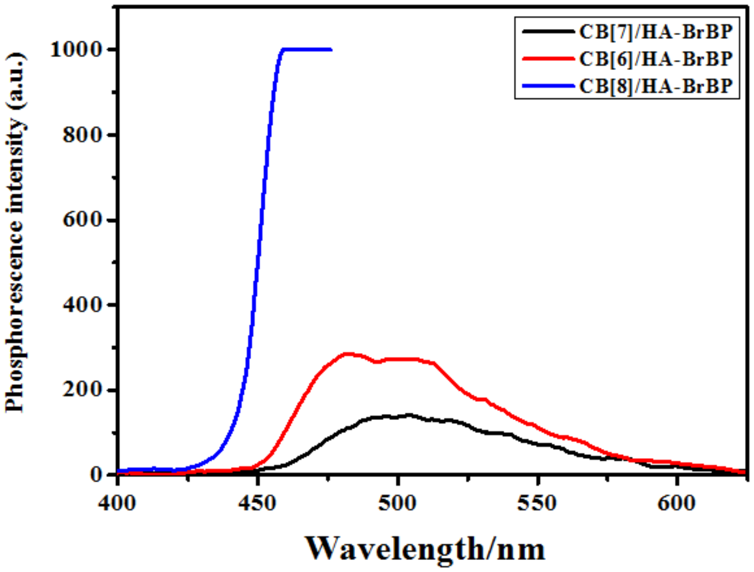

Supplementary Figure 8. Effect of complexation with CB[6], CB[7] and CB[8] on the spectra of HA–BrBP. The phosphorescence spectra of CB[6]/HA–BrBP, CB[7]/HA–BrBP and CB[8]/HA–BrBP at 298 K in aqueous solution (delayed by 0.2 ms, Ex. Slit = 10 nm, Em. Slit = 10 nm). ([BrBP] = 0.5 mM, [CB[6]] = 0.5 mM, [CB[7]] = 0.5 mM, [CB[8]] = 0.25 mM).

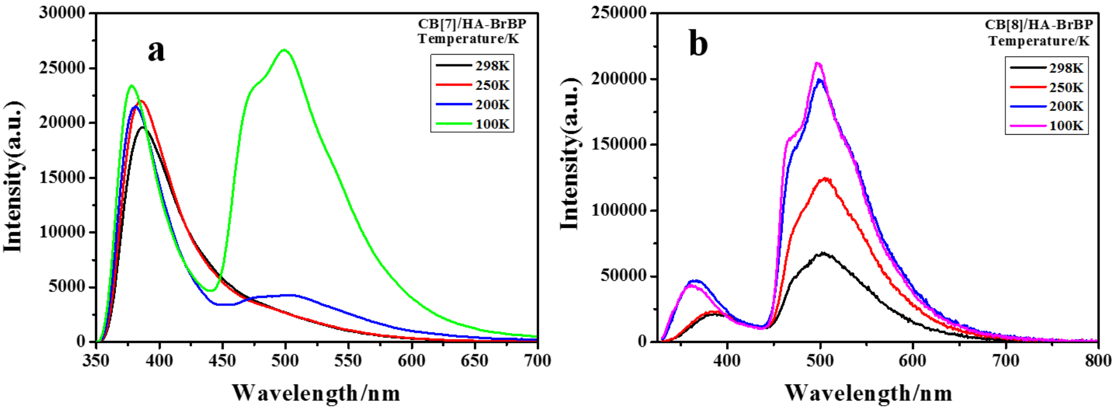

Supplementary Figure 9. Temperature effect on the photoluminescence spectra of CB[7, 8]/HA–BrBP. Prompt photoluminescence spectra of (a) CB[7]/HA–BrBP and (b) CB[8]/HA–BrBP in the temperature range from 298 K to 100 K in aqueous solution.

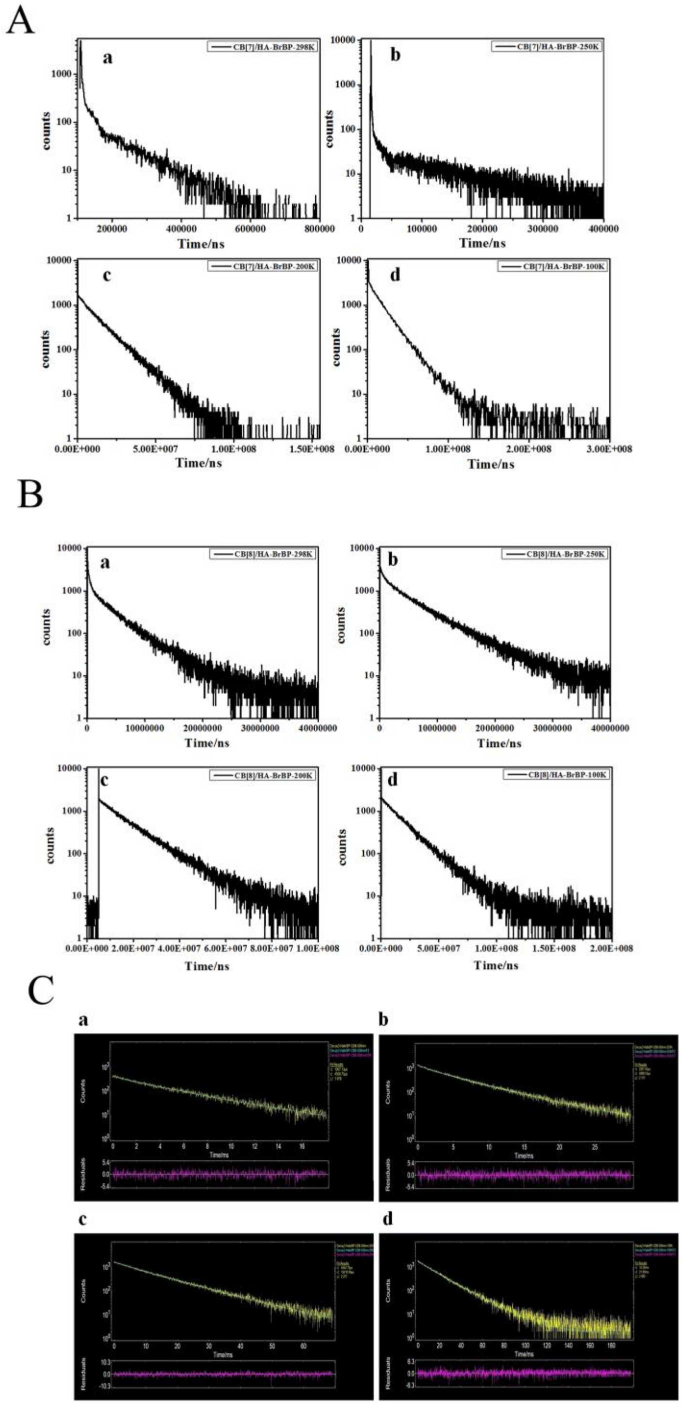

Supplementary Figure 10. Variable–temperature phosphorescence lifetime contrast curves for CB[7]/HA–BrBP and CB[8]/HA–BrBP. (**A**) The Phosphorescence lifetime decay curve of CB[7]/HA–BrBP for 500 nm from (a) 298 K; (b) 250 K; (c) 200 K to (d) 100 K. ([BrBP] = 0.5 mM, [CB7] = 0.5 mM); (**B**) The Phosphorescence lifetime decay fitting curve of CB[8]/HA–BrBP for 500 nm from (a) 298 K; (b) 250 K; (c) 200 K to (d) 100 K.

([BrBP] = 0.5 mM, [CB8] = 0.25 mM); (**C**) The Phosphorescence lifetime decay fitting curve of CB[8]/HA–BrBP for 500 nm from (a)298 K; (b) 250 K; (c) 200 K to (d) 100 K. ([BrBP] = 0.5 mM, [CB8] = 0.25 mM).

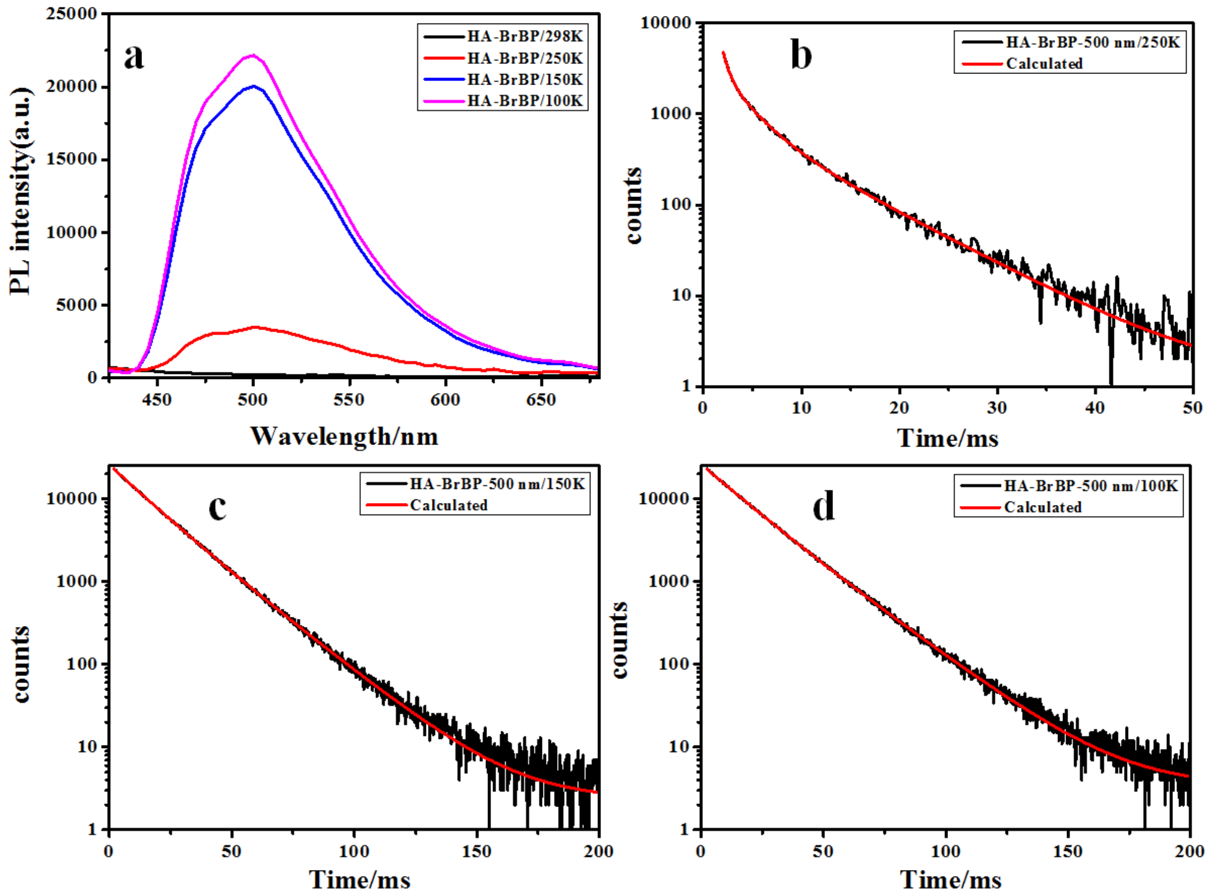

Supplementary Figure 11. Temperature effect on the photoluminescence spectra and lifetime curves of HA–BrBP. (**a**) Prompt photoluminescence spectra of HA–BrBP in the temperature range from 298 K to 100 K in aqueous solution; The Phosphorescence lifetime decay fitting curve of HA–BrBP for at 500 nm from (b) 250 K; (c) 150 K to (d) 100 K. ([BrBP] = 0.5 mM).

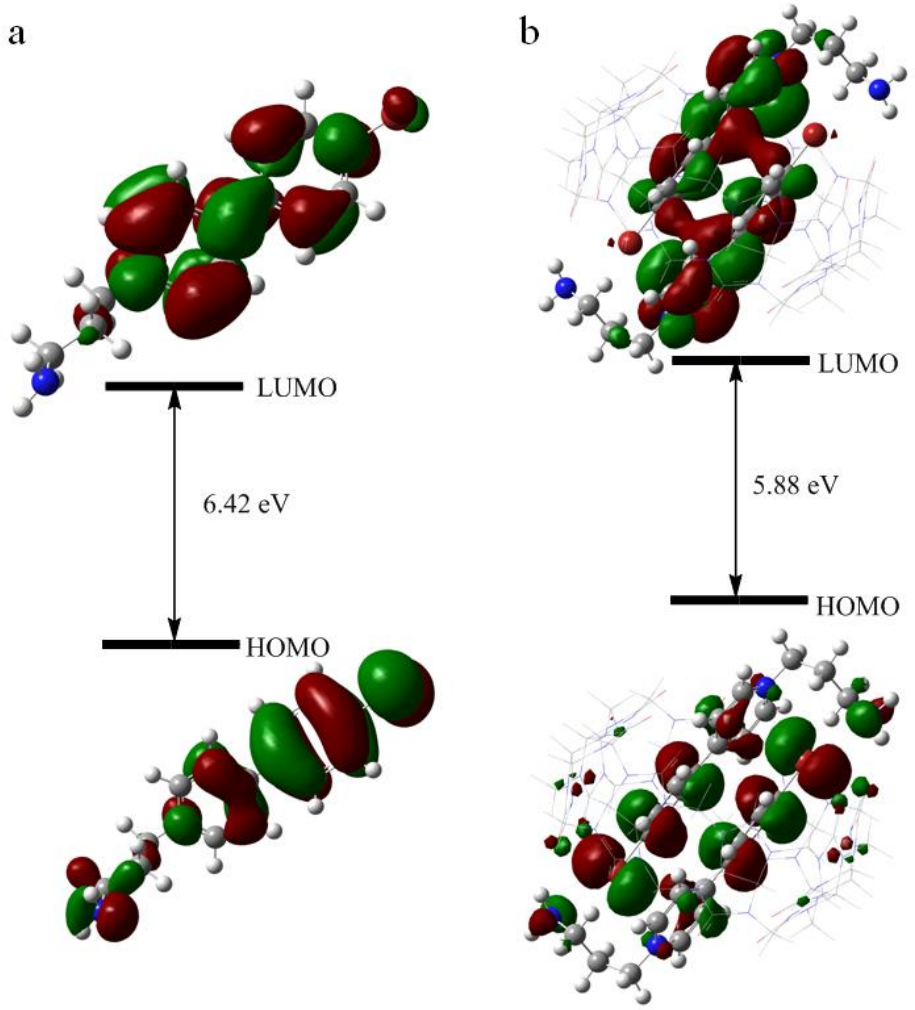

Supplementary Figure 12. Frontier molecular orbitals and energy gaps. (**a**) FMOs and energy gaps of the single guest (SPE= –3226.540604 a.u.); (**b**) FMOs and energy gaps of the CB[8]/BrBP–NH_2 (SPE= –11267.065376 a.u.).

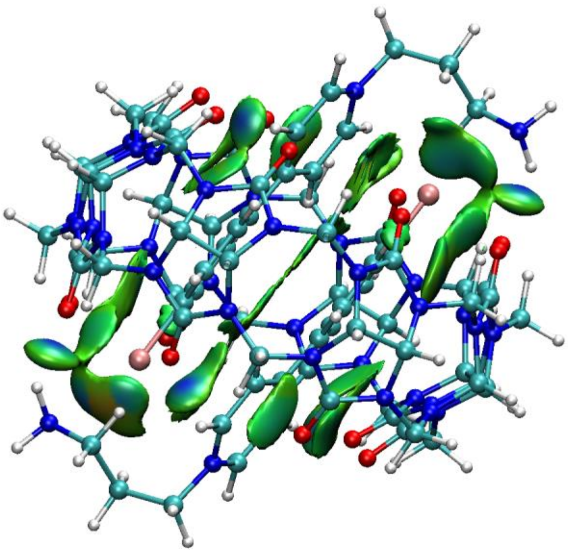

Supplementary Figure 13. The non-covalent interaction (NCI) analysis. The non-covalent interaction (NCI) analysis of two same guest molecules and CB[8].(Isosurface value=0.01. The green, blue, and red regions represent the weak, strong, and repulsive interactions, respectively.)

Supplementary Table 2. Some key Mulliken charge of single molecule and assembly in water at M06-2X-GD3/6-311G(d,p) level.

	Number-Atom	Charge in single molecule	Charge in assembly
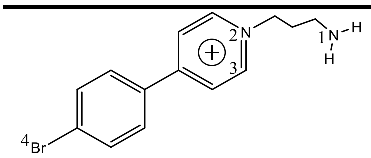	1-N	-0.585	-0.558
	2-N	-0.393	-0.360
	3-C	0.223	0.175
	4-Br	0.003	0.063

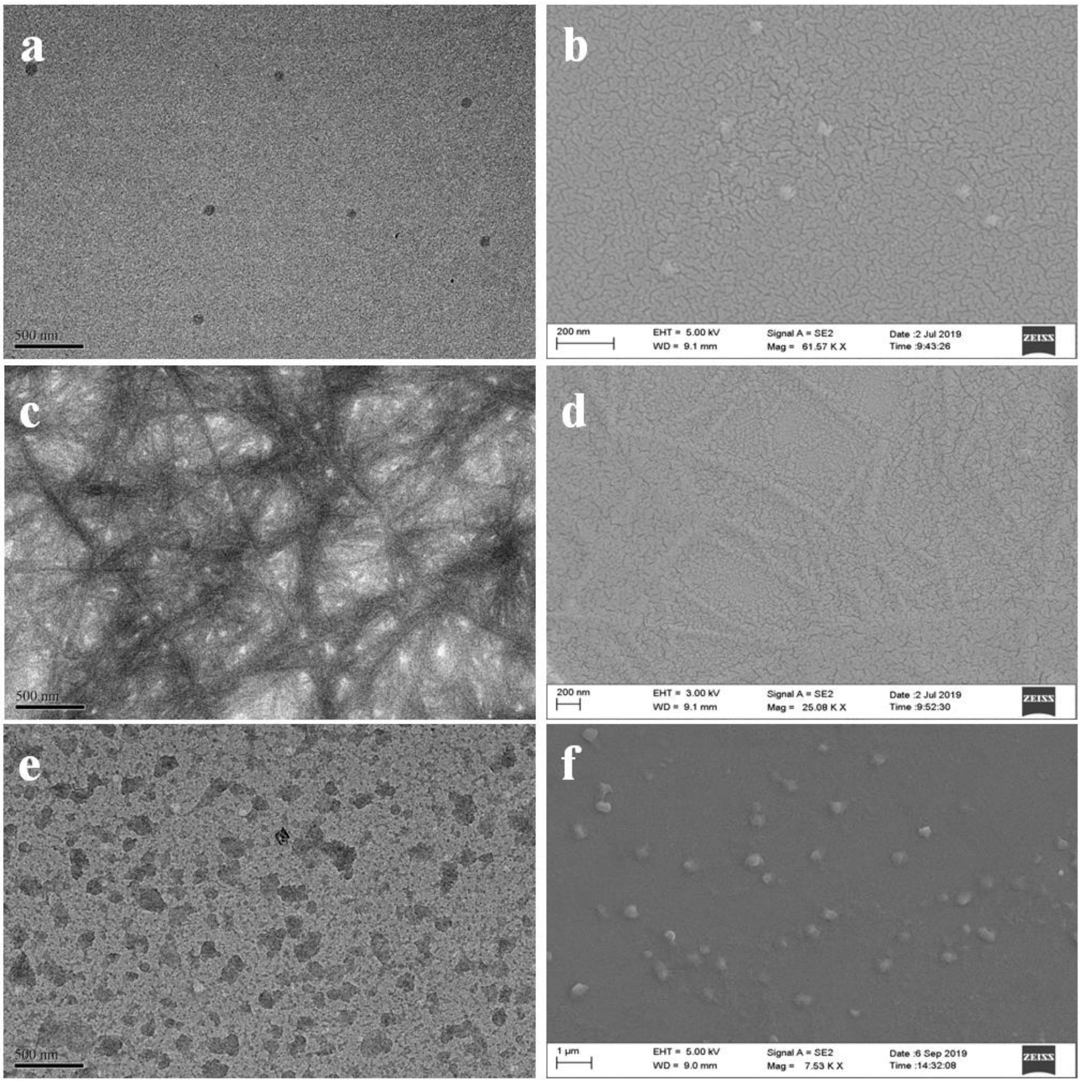

Supplementary Figure 14. Transmission and scanning electron microscopy images. TEM image of (**a**) HA–BrBP, (**c**) CB[7]/HA–BrBP, (**e**) CB[8]/HA–BrBP and SEM image of (**b**) HA–BrBP, (**d**) CB[7]/HA–BrBP, (**f**) CB[8]/HA–BrBP.

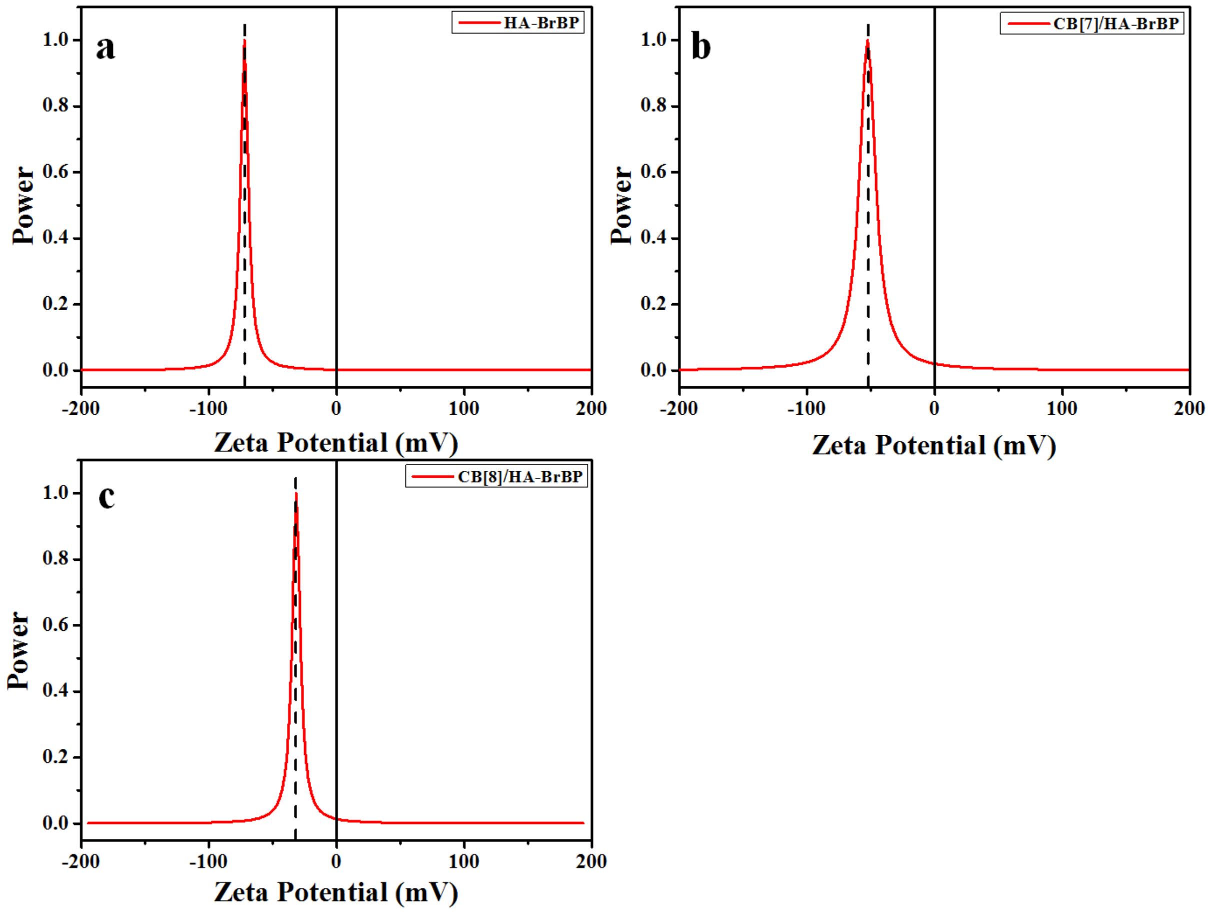

Supplementary Figure 15. Zeta potential data. Zeta potential results of (**a**) HA–BrBP (**b**) CB[7]/HA–BrBP and (**c**) CB[8]/HA–BrBP.

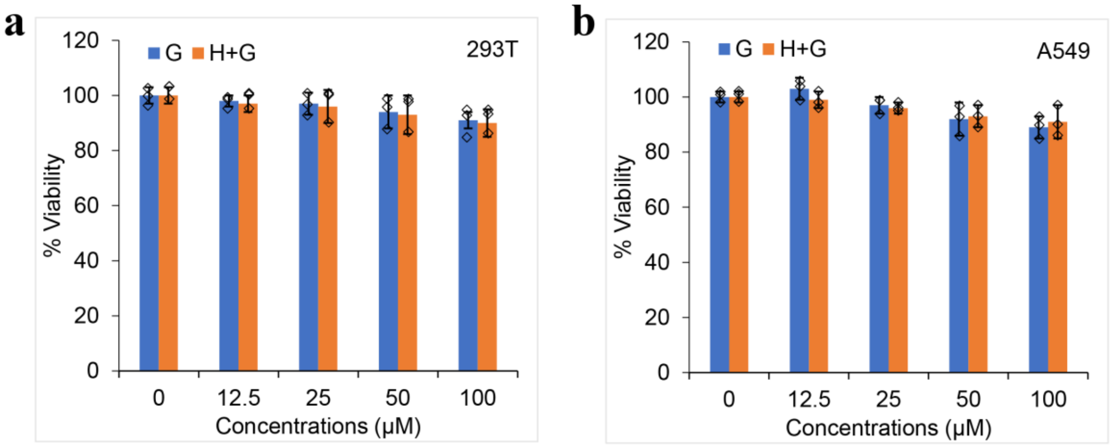

Supplementary Figure 16. Cytotoxicity assay results for HA–BrBP and CB[8]/HA–BrBP. Relative cell viability of HA–BrBP(G) and CB[8]/HA–BrBP(H+G) with different concentrations([BrBP]= 0 μM, 12.5 μM, 25 μM, 50 μM, 100 μM) at 25 ℃ in (**a**) 293T cells, (**b**) A549 cells. n = 3 independent experiments, with the bar data indicating mean ± SD. Noting that there is no significant difference between the G group and the H+G group under each concentration for both 293T and A549 cells ($P < 0.05$).

a

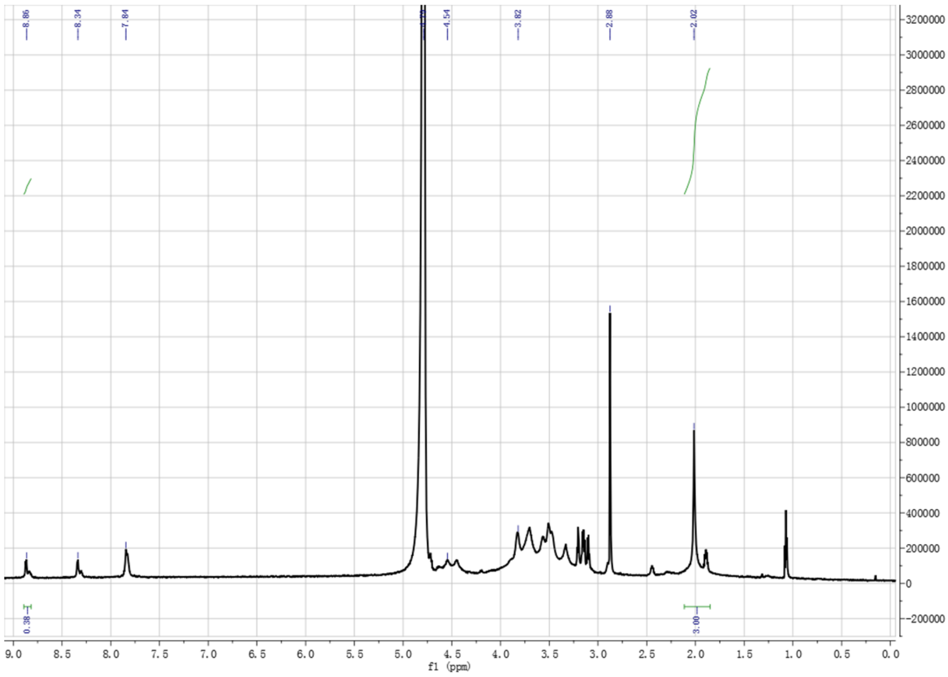
8.86
8.34
7.84
4.54
3.82
2.88
2.02
0.38
3.00
f1 (ppm)

b

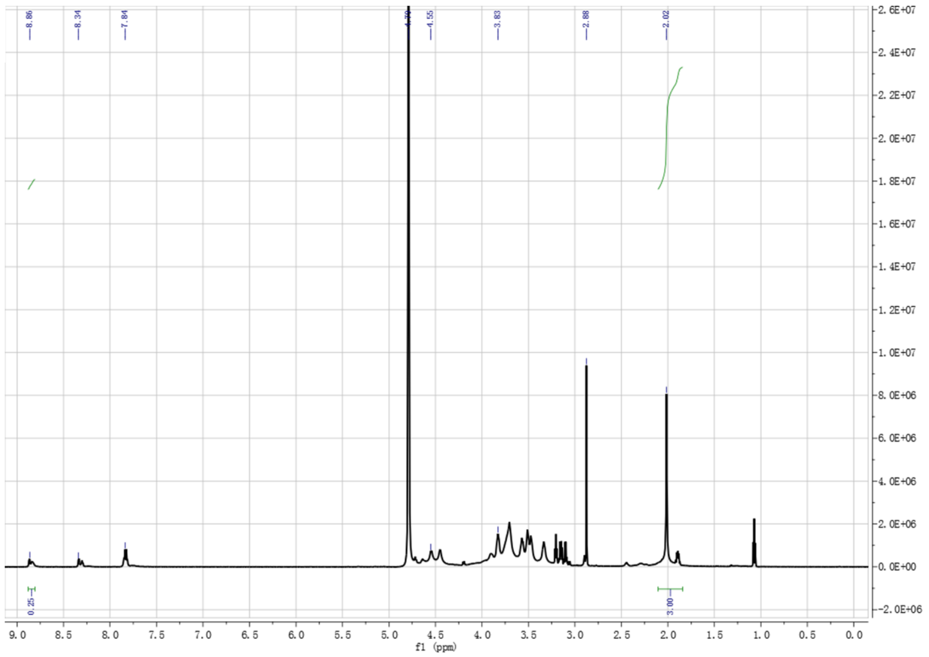
8.86
8.34
7.84
4.55
3.83
2.88
2.02
0.25
3.00
f1 (ppm)

c

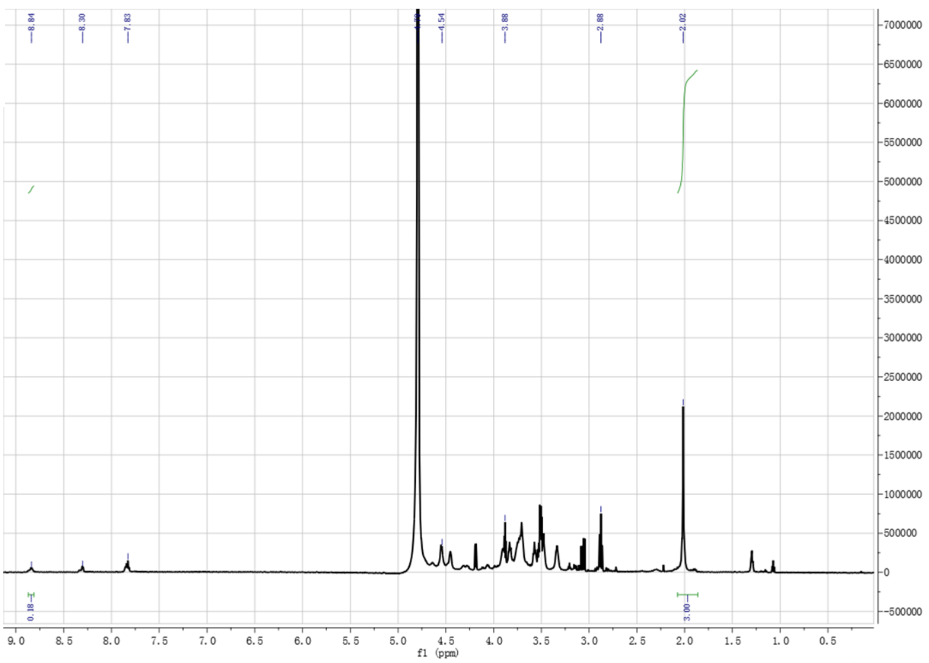

Supplementary Figure 17. ^{1}H NMR spectrum of different molecular weight HA modified BrBP. ^{1}H NMR spectrum of (**a**) HA_{3k}–BrBP (molecular weight of HA for 3k) (**b**) HA_{10w}–BrBP (molecular weight of HA for 100k) and (**c**) HA_{1000k}–BrBP (molecular weight of HA for 1000k) in D_2O at 298 K.

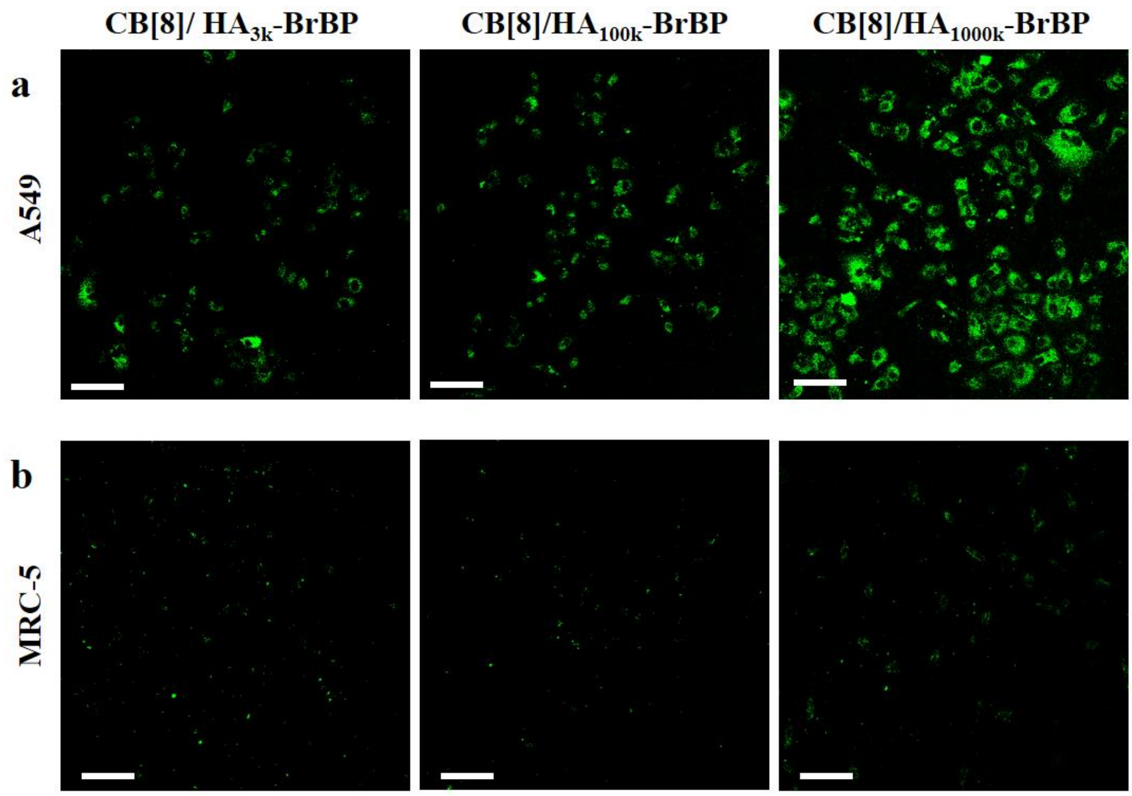

Supplementary Figure 18. Confocal microscopy images of different molecular weight HA modified BrBP. (**a**) A549 cells incubated with HA_{3k}–BrBP, HA_{100k}–BrBP and HA_{1000k}–BrBP. (**b**) MRC–5 cells incubated with HA_{3k}–BrBP, HA_{100k}–BrBP and HA_{1000k}–BrBP ([BrBP] = 25 μM). (Scale bar = 100 μm)

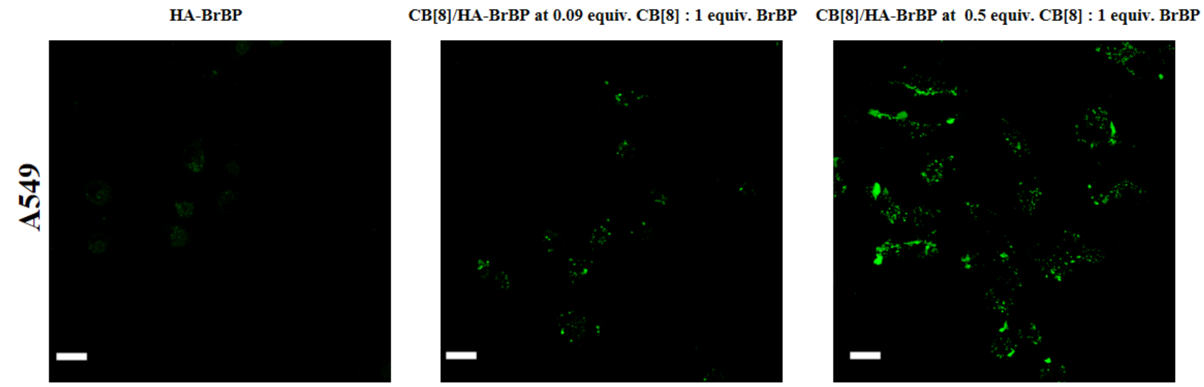

Supplementary Figure 19. Confocal microscopy images of different amount of CB[8] on for the HA–BrBP. A549 cells incubated with HA–BrBP, 0.09 equiv CB[8]/HA–BrBP and 0.5 equiv CB[8]/HA–BrBP ([BrBP] = 25 μM). (Scale bar = 30 μm)

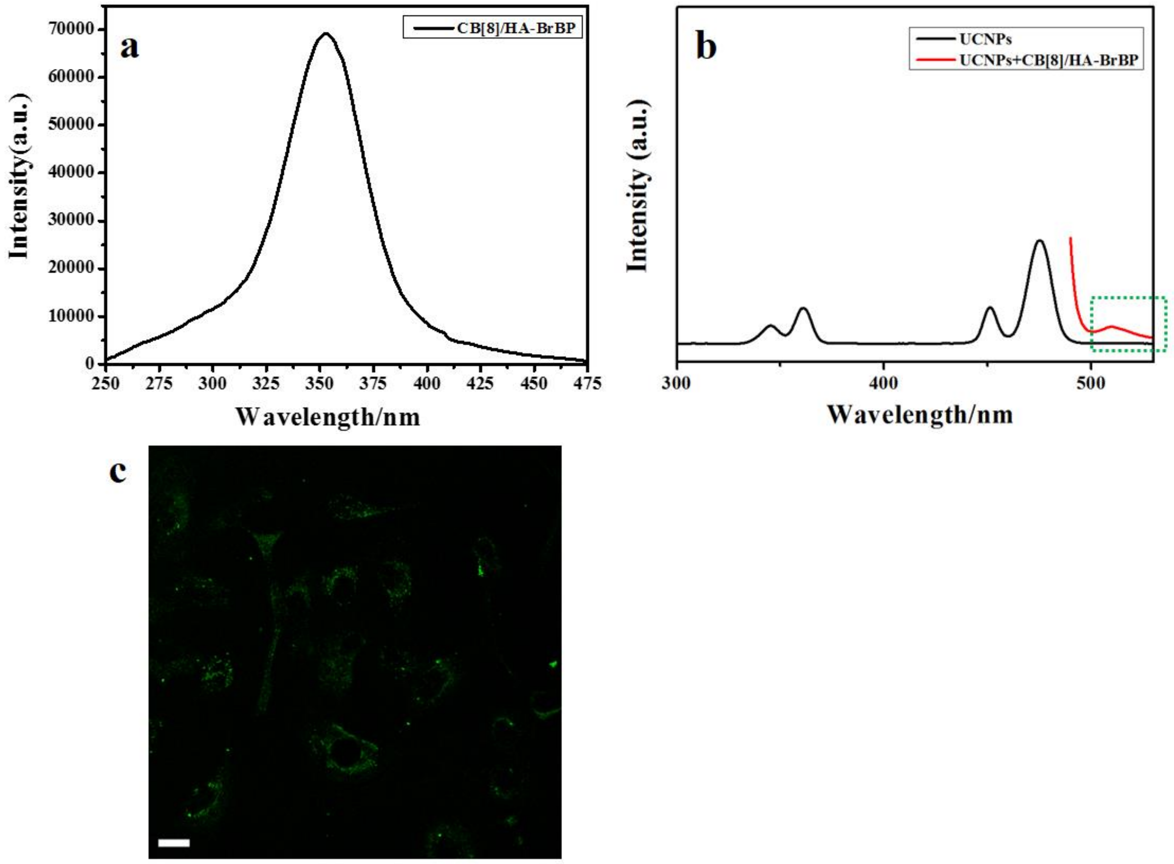

Supplementary Figure 20. The Prompt photoluminescence contrast spectra of UCNPs and UCNPs/CB[8]/HA–BrBP. (**a**) The excitation spectra of the CB[8]/HA–BrBP upon photoirradiation in aqueous solution at 298 K. (**b**) The prompt photoluminescence contrast spectra of UCNPs (black) and UCNPs/CB[8]/HA–BrBP (red) in water (298 K, λ_{ex} = 980 nm). (**c**) Confocal microscopy images of HeLa cells incubated with UCNPs/CB[8]/HA–BrBP (λ_{ex} = 488 nm). UCNPs (7.5 mg/mL, $NaYREF_4$, RE: Yb, Er, Tm, Gd, Mu, Lu) was purchased from Hefei Fluonano Biotech Co., Ltd. (Scale bar = 30 μm)
