# Supplementary material for: Ultralong purely organic aqueous phosphorescence supramolecular polymer for targeted tumor cell imaging
Source: Nat Commun. 2020 Sep 16;11:4655. doi: 10.1038/s41467-020-18520-7 (PMC7494876; doi:10.1038/s41467-020-18520-7)
Supplement: Supplementary file 3 — Reporting Summary [file 41467_2020_18520_MOESM3_ESM.pdf]

nature research

Corresponding author(s): yuliu

Last updated by author(s): Aug 20, 2020

# Reporting Summary

Nature Research wishes to improve the reproducibility of the work that we publish. This form provides structure for consistency and transparency in reporting. For further information on Nature Research policies, see our Editorial Policies and the Editorial Policy Checklist.

## Statistics

For all statistical analyses, confirm that the following items are present in the figure legend, table legend, main text, or Methods section.

| n/a | Confirmed | |
|---|---|---|
| ☐ | ☒ | The exact sample size ($n$) for each experimental group/condition, given as a discrete number and unit of measurement |
| ☐ | ☒ | A statement on whether measurements were taken from distinct samples or whether the same sample was measured repeatedly |
| ☐ | ☒ | The statistical test(s) used AND whether they are one- or two-sided<br>*Only common tests should be described solely by name; describe more complex techniques in the Methods section.* |
| ☐ | ☒ | A description of all covariates tested |
| ☒ | ☐ | A description of any assumptions or corrections, such as tests of normality and adjustment for multiple comparisons |
| ☐ | ☒ | A full description of the statistical parameters including central tendency (e.g. means) or other basic estimates (e.g. regression coefficient) AND variation (e.g. standard deviation) or associated estimates of uncertainty (e.g. confidence intervals) |
| ☐ | ☒ | For null hypothesis testing, the test statistic (e.g. $F$, $t$, $r$) with confidence intervals, effect sizes, degrees of freedom and $P$ value noted<br>*Give P values as exact values whenever suitable.* |
| ☒ | ☐ | For Bayesian analysis, information on the choice of priors and Markov chain Monte Carlo settings |
| ☒ | ☐ | For hierarchical and complex designs, identification of the appropriate level for tests and full reporting of outcomes |
| ☒ | ☐ | Estimates of effect sizes (e.g. Cohen's $d$, Pearson's $r$), indicating how they were calculated |

*Our web collection on statistics for biologists contains articles on many of the points above.*

## Software and code

Policy information about availability of computer code

| | |
|---|---|
| Data collection | UVProbe 2.21, VPViewer 2000 ITC, F980, Omega_9.0.33, TopSpin 3.2, DigitalMicrograph 2.02.800.0, SmartSEM V05.07, Leica Application Suite X, Particle Solutions v. 2.6 |
| Data analysis | Microsoft Excel 2013, Gaussian 16, SPSS 20.0, OriginPro 2020b(Learning Edition), CYLView 1.0b, Multiwfn 3.7, VMD 1.9.3 |

For manuscripts utilizing custom algorithms or software that are central to the research but not yet described in published literature, software must be made available to editors and reviewers. We strongly encourage code deposition in a community repository (e.g. GitHub). See the Nature Research guidelines for submitting code & software for further information.

## Data

Policy information about availability of data

All manuscripts must include a data availability statement. This statement should provide the following information, where applicable:

- Accession codes, unique identifiers, or web links for publicly available datasets
- A list of figures that have associated raw data
- A description of any restrictions on data availability

We ensure that all data in this study are available in the paper and in its SI, and supplied the "Data Availability" section in the Methods:"Data availability. The authors declare that the data supporting the findings of this study are available within the paper and its Supplementary Information. All data are available from the authors on reasonable request."

# Field-specific reporting

Please select the one below that is the best fit for your research. If you are not sure, read the appropriate sections before making your selection.

- [x] Life sciences
- [ ] Behavioural & social sciences
- [ ] Ecological, evolutionary & environmental sciences

For a reference copy of the document with all sections, see nature.com/documents/nr-reporting-summary-flat.pdf

# Life sciences study design

All studies must disclose on these points even when the disclosure is negative.

| | |
|---|---|
| Sample size | All cell culture experiments were performed in triplicates to allow for calculation of standard deviation standard errors of the mean and t-statistics for use in two-side Student's t-test. |
| Data exclusions | no data were excluded from the analysis. |
| Replication | All experiments were performed with at least three technical replicates on more than one occasion to ensure reproducibility across experiments. |
| Randomization | Randomization was not relevant to our study design as we investigated single factors within each study. |
| Blinding | Blinding was not relevant as all processing methods were done with consistent parameters utilized across all treatment groups. |

# Reporting for specific materials, systems and methods

We require information from authors about some types of materials, experimental systems and methods used in many studies. Here, indicate whether each material, system or method listed is relevant to your study. If you are not sure if a list item applies to your research, read the appropriate section before selecting a response.

## Materials & experimental systems

| n/a | Involved in the study | |
|---|---|---|
| x | | Antibodies |
| | x | Eukaryotic cell lines |
| x | | Palaeontology and archaeology |
| x | | Animals and other organisms |
| x | | Human research participants |
| x | | Clinical data |
| x | | Dual use research of concern |

## Methods

| n/a | Involved in the study | |
|---|---|---|
| x | | ChIP-seq |
| x | | Flow cytometry |
| x | | MRI-based neuroimaging |

## Eukaryotic cell lines

Policy information about cell lines

| | |
|---|---|
| Cell line source(s) | A549, HeLa, KYSE–150, 293T, and MRC–5 were purchased directly from the Cell Resource Center of China Academy of Medical Science, Beijing, China. |
| Authentication | No extra authentication was performed for the cell lines directly purchased from the Cell Resource Center of China Academy of Medical Science. |
| Mycoplasma contamination | All cell lines tested negative for mycoplasma contamination. |
| Commonly misidentified lines (See ICLAC register) | No commonly misidentified lines were used. |
